# Transition Metals in Freshwater Crustaceans, Tilapia, and Inland Water: Hazardous to the Population of the Small Island Province

**DOI:** 10.3390/toxics9040071

**Published:** 2021-04-01

**Authors:** Christine Joy M. Agarin, Doreen R. Mascareñas, Ronnel Nolos, Eduardo Chan, Delia B. Senoro

**Affiliations:** 1 School of Graduate Studies, Mapua University, Manila 1002, Philippines; christinejoyagarin@gmail.com; 2 School of Chemical, Biological, Materials Engineering and Sciences, Mapua University, Manila 1002, Philippines; 3 Yuchengco Innovation Center, Mapua University, Manila 1002, Philippines; doreen.mascarenas@g.msuiit.edu.ph (D.R.M.); ronnelnolos@gmail.com (R.N.); 4 School of Agriculture, Marinduque State College, Torrijos, Marinduque 4903, Philippines; 5 Mapua-MSC Joint Research Laboratory, Marinduque State College, Boac, Marinduque 4900, Philippines; 6 Dyson College of Arts and Sciences, Pace University, New York, NY 10038, USA; echan@pace.edu; 7 School of Civil, Environmental, and Geological Engineering, Mapua University, Manila 1002, Philippines

**Keywords:** fishes, inland water, metals, risks, island, spatial distribution, toxicants

## Abstract

This paper elaborates on the potential toxicants detected in inland water, freshwater crustaceans, and tilapia in an island that experienced mining disasters in 1993 and 1996. Specimen samples were collected in six municipalities of the island province in 2019 and presence of metals (Cd, Cr, Cu, Fe, Mn, Ni, Pb, and Zn) were analyzed using Inductively Coupled Plasma-Optical Emission Spectrometer (ICP-OES). Potential ecological risks analysis followed the Hakanson approach. Canonical correspondence analysis PAST Version 3.22, IBM SPSS 25.0, and Pearson correlation were employed for statistical analysis, and GIS Pro 2.5 for mapping of sampling locations and spatial distribution. Results showed that Mn and Zn concentration was highest in surface water (SW) and groundwater (GW), respectively. All metal concentration values exceeded the maximum permissible limit by regulatory international organizations. Elevated concentration of Cr, Cu, Fe, Mn, and Zn was detected in both crustaceans and tilapia. The calculated health hazard indices were greater than one, which means potential high adverse effects on public health when ingested. The municipality of Sta. Cruz and Torrijos recorded higher potential ecological risk among the six municipalities. Results of the correlation analysis suggested that metals in SW and GW have a similar origin, mutual dependence, and identical behavior during transport.

## 1. Introduction

The province of Marinduque is a small island in the Philippines located at 13.4767° N, 121.9032° E. It has a land area of 959.25 km^2^, is about 170 km south of Metro Manila, and has six municipalities, namely, Boac (B), Buenavista (BV), Gasan (G), Mogpog (M), Torrijos (T), and Sta. Cruz (S), and 218 barangays. Barangays are the smallest territorial, administrative and local level of government unit. Marinduque has a population of 234,521 and its longest river is approximately 27 km, recorded to have the largest copper reserves in the country [1], and one of the world’s largest copper mines during the period of 1969–1996 [2]. This contributed to the local economy of the island. The activities engaged in open pit mining in the municipality of Boac and Sta. Cruz in the early 1970s started at the portion of Mt. Tapian, Boac ore deposit. The mine wastes from Tapian pit were discharged to Calancan Bay, Sta. Cruz through a drainage tunnel. When the ore deposits at Mt. Tapian were depleted in late 80s, the mining operation moved to Maguilaguila, Sta. Cruz in 1990. This new site is about three kilometers north of Tapian with its own tailings/siltation pond impounded by an earth dam. The depleted Tapian open pit was used as temporary storage of mine wastes from Maguilaguila. On 6 December 1993, the Maguilaguila siltation dam collapsed and mine tailings flux was hosted by Mogpog River. Riverside barangays were flooded with mine tailings. On 24 March 1996, i.e., two years and three months after the Maguilaguila mining disaster event, Tapian dam failed and the 27 km long, Boac River was flooded with mine tailings [3]. Mogpog and Boac are adjacent municipalities. Mining activities were halted in 1997 leaving two abandoned mine pits. One pit has an opening of two kilometers long. The loss of riverine habitat, including the substantial increase in the magnitude of flood events after the two disaster events were associated with, according to the local population, the 1993 and 1996 mining disaster events [1].

Mine tailings contain pyrite (iron sulfide) or chalcopyrite (copper-iron sulfide). Sulfidic mine tailings are prone to produce acid mine drainage, which has been reported to cause adverse effects to the environment, aquatic life, and public health [4]. Further, open pit mining exposes the subsurface walls to moisture and the atmospheric oxygen speeds up the oxidation process by anaerobic bacteria (*Thiobacillus ferrooxidans*). In addition, the open pit stores water during heavy precipitation or typhoon, which increases the rate of oxidation by a factor of one million [5]. The iron sulfides in aquatic environment are hazardous and classified as an acute hazard, Category 1, H400 by the European Union REACH (Registration, Evaluation, Authorization and Restriction of Chemicals) under the CLP Regulation (EC) No. 1272/2008. The oxidation process acidifies the environment and mobilizes metals such as lead (Pb), arsenic (As), cadmium (Cd), and among other metals. Exposed rocks or subsurface walls of open mine pits produce acid rock drainage and are regarded as autocatalytic due to feedback process, which is difficult to control [5]. Metals released and mobilized by the technological activities of humans tend to persist indefinitely, circulating and accumulating throughout the food chain.

At the right concentration, many metals are essential to life. However, in excess or elevated concentration, same metals can be considered toxicants. Toxic heavy metals residues in the environment, if not controlled and managed well, can be hazardous to the public. Food chains are affected when mining disaster happens; hence, other countries require screening of chemicals in fish and fishery products [6], especially if fishes and crustaceans are part of the population’s diet. However, regular screening of metals in fish and other agricultural products has not been a regular practice and not part of a regulation in the Philippines. According to Baby et al. [7], heavy metals become toxic if not metabolized by the body and accumulate in the soft tissues. The low-level chronic exposure to heavy metals not metabolized and discharge by the body become a public health problem.

There were various studies focusing on river and coastal water quality, sediments, and human health in Marinduque [8,9,10,11,12] and neighboring countries [13,14,15,16]. However, there was very limited study on metal detection in freshwater crustaceans and tilapia (fish) and the spatial distribution of the potential ecological risks (PER) posed by these freshwater fish together with inland water quality. Hence, this study focused on understanding the inland and freshwater tilapia and crustacean’s quality, and the potential risks when ingested by the population. Information on the spatial distribution of metals pollution and its ecological risks is helpful in the local and national government in making relevant strategies, programs, and policies to protect the environment and the public.

## 2. Materials and Methods

### 2.1. Study Area and Sample Collection

**Figure 1:**
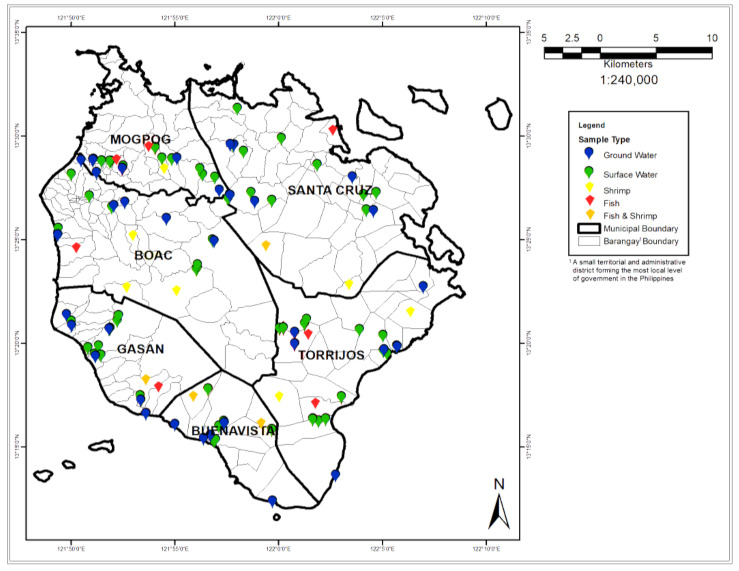
Map of the study area and location of sampling points.

Commercially available freshwater crustaceans (*Macrobrachium* sp.) and tilapia (*Oreochromis niloticus* and *Channa striata*) were bought from local fishermen. Surface water (SW) and groundwater (GW) samples were collected across the Marinduque island (Figure 1). Marinduque, Philippines is a tropical and warm island with an annual mean temperature of 27 °C. The island province sets on volcanic, igneous, and sedimentary rocks. These media are porous and groundwater can flow through porous media. There is no pronounced wet and dry season in the island as precipitation is experienced throughout the year. Criteria for selection of freshwater fishes and crustaceans were based on what was most often eaten by people living on the island. The collected samples were packed in a polyethylene container, sealed, labeled, and stored in a cooler at approximately 4 °C before transferring to the laboratory for treatment and analysis. The SW and GW samples were collected in accordance with the U.S. EPA Number SESDPROC-201-R4 and U.S. EPA and SESDPROC-301-R4 protocol for SW and GW sampling, respectively. The source of water for fishponds are normally from shallow wells. Crustaceans were collected from rivers and streams. Size of crustaceans was considered during collection to acquire sufficient mass after drying for analysis.

### 2.2. Sample Preparation for Heavy Metal Analysis

Twenty four (24) tilapia and crustaceans, sixty-two (62) and thirty-five (35) samples of SW and GW, respectively, were collected in 2019 at various locations described in Figure 1. Crustaceans and tilapia were collected in 11 and 13 different places in Marinduque, respectively. The samples were cut into about 0.5 cm pieces and dried at MSC-Mapua joint research laboratory at Boac, Marinduque using a dehydrator at 68 °C for 7–8 h. A total of 121 samples were analyzed in triplicates for the presence of eight transition and post-transition metals such as cadmium (Cd), chromium (Cr), copper (Cu), iron (Fe), lead (Pb), manganese (Mn), nickel (Ni), and zinc (Zn) using Inductively Coupled Plasma Optical Emission Spectrometer (ICP-OES) Perkin Elmer Optima 8000 with minimum R^2^ = 0.9995 per metal. The digestion and analysis were in accordance with U.S. EPA Method 200.3/200.11 and U.S. EPA Method 3005A/200.7 for tilapia and crustaceans’ tissues and inland water samples, respectively. The analysis was carried out at the Wet Laboratory of Yuchengco Innovation Center of Mapua University in Manila, Philippines.

### 2.3. Potential Ecological Risk Determination

The data collected from ICP-OES were used to calculate the pERI following the Håkanson’s sedimentological approach [17]. This requires four hypotheses to determine the potential ecological risk index (pERI) such as: (a) concentration–pERI increases as concentration increases; (b) number of pollutants–the pERI increases as the number of target metals increases; (c) toxic factor–pERI shall be graded; and (d) sensitivity–various inland waters have different sensitivity to toxic substance. Based on these hypotheses, this study used Equations (1) and (2), as shown below.

(1) $$C_{d}=\;\overset{8}{\underset{i=1}{\sum }}C_{f}^{i}=\;\overset{8}{\underset{i=1}{\sum }}\frac{C_{0}^{i}}{C_{n}^{i}}$$

(2) $$pERI=\;\overset{8}{\underset{i=1}{\sum }}E_{r}^{i}=\;\overset{8}{\underset{i=1}{\sum }}T_{r}^{i}.C_{f}^{i}$$

where, *C_d_* is the degree of contamination or the concentration of the metal (*i*); $C_{f}^{i}$ is the contamination factor of metal; $C_{0}^{i}$ is the mean concentration of the metal in water, tilapia, and crustaceans; $C_{n}^{i}$ is the standard pre-industrial reference concentration level; *pERI* is the potential ecological risk index of water, tilapia, and crustaceans, which is the sum of $E_{r}^{i}$. The $E_{r}^{i}$ is the potential ecological risk factor for metal; and $T_{r}^{i}$ is the toxic-response factor/coefficient for metals, i.e., Cd = 30, Cr = 2, Cu = 5, Pb = 5, and Zn = 1 [17]. The PER represents the sensitivity of a biological community to toxic substances caused by the pollution/contamination, which can be used as a diagnostic tool for water pollution control. This pERI determination approach categorized the contamination factor and degree of contamination into four groups as shown in Table 1. While values for background reference $\left(C_{n}^{i}\right)$ and toxicity coefficients $\left(T_{r}^{i}\right)$ of metals, shown in Table 2, necessary in calculating the $\left(C_{f}^{i}\right)$ and $E_{r}^{i}$, respectively. The PER is expressed in terms of a comprehensive pERI, which is the sum of all ($E_{r}^{i}$)s. The total PER includes the determination of the carcinogenicity of the metals to human when ingested, as illustrated in Equations (3)–(7).

The potential human health risk (pHHR) index from the consumption of tilapia, crustaceans, and ingestion of water were estimated by calculating the estimated daily intake (EDI), target hazard quotient (THQ), and carcinogenicity risk (CR) all in accordance with the U.S. EPA [18,19]. The level of exposure through ingestion/oral route of a particular heavy metal in tilapia/crustaceans and water were expressed by calculating the EDI using Equations (3) and (4), respectively.

(3) $$EDI=\frac{C_{m}\;\cdot \;E_{f}\;\cdot \;E_{d}\;\cdot \;IR\;\cdot \;CF_{}}{BW\;\cdot \;AT_{n}}\;\times \;10^{-3}$$

(4) $$EDI=\frac{C_{m}\;\cdot \;E_{f}\;\cdot \;E_{d}\;\cdot \;IR}{BW\;\cdot \;AT_{n}}$$

where $C_{m}$ is the concentration of the metals in the samples (mg kg^−1^ or mg L^−1^); IR is the ingestion rate (g day^−1^ or L day^−1^); $E_{f}$ is exposure frequency (365 d yr^−1^); $E_{d}$ is the exposure in years; BW is the average adult body weight (kg); $AT_{n}$ is the averaging time; *CF* is the conversion factor.

The THQ, used to investigate the risk of non-carcinogenic effects, is the ratio between the EDI and the oral reference dose [RfD_o_, (mg kg^−1^ day^−1^)^−1^], and was calculated using Equation (5) [18,20,21].

(5) $$THQ=\;\frac{EDI}{RfD_{o}}$$

Since exposure to two or more pollutants may result in additive and/or interactive effects, cumulative health risk, or the health hazard index (HHI), can be evaluated by summing the individual metal target hazard quotient values and was calculated using Equation (6) [20,21,22].

(6) $$HHI=\;THQ_{\left(toxicant\;1\right)}+\;THQ_{\left(toxicant\;2\right)}+\ldots +\;THQ_{\left(toxicant\;n\right)}\;$$

When the calculated THQ/HHI value is <1, adverse health effects to the concerned receptors are not likely to occur. If THQ/HHI = 1, non-carcinogenic health risk is likely. However, should THQ/HHI >1, the metal concentration exceeds the reference concentration [18]. The ingestion dose in this consideration was assumed to be equal to the absorbed contaminant dose considering that cooking has no effect on the concentrations of metals.

Carcinogenic risk assessment evaluates the likelihood of an individual developing cancer due to exposure to the potential carcinogen over a lifetime [23]. Equation (7) [18] was used for the estimation of the cancer risk (CR) using the carcinogenicity potency factor [or cancer slope factor, CSF, (mg kg^−1^ day^−1^)^−1^] and the EDI from equation for tilapia and crustaceans and Equation (4) for SW and GW.

(7) CR=CSF · EDI

### 2.4. Statistical Analysis and Spatial Distribution

Canonical correspondence analysis (CCA) was employed using PAST Version 3.22 software to determine the relationship of the PERs with HHRs. This was coupled with the IBM SPSS Statistics Version 25.0 and Pearson correlation coefficient analysis. After which, GIS Pro Version 2.5 was employed to plot the sampling locations and mapped the spatial distribution of metal concentration in water and the potential ecological risks (PER) across the island province.

## 3. Results

Subsections below elaborate on the toxicants detected from freshwater crustaceans (*Macrobrachium* sp.), tilapia (*O. niloticus* and *C. striata*), and SW and GW. It further illustrates the spatial distribution of these toxicants and its potential ecological risk as well as its trend.

### 3.1. Concentration of Metals in Surface Water and Groundwater

The SW samples had concentrations of Cd (0–0.032), Cr (0–0.016), Cu (0–2.454), Fe (0.005–0.846), Pb (0–0.032), Mn (0.014–2.450), Ni (0–0.043), and Zn (0–1.651) mgL^−1^. Metal concentrations in GW ranged from Cd (0.001–0.099), Cr (0.017–0.113), Cu (0.001–0.121), Fe (0.482–10.70), Pb (0.002–0.117), Mn (0.098–1.483), Ni (0.001–0.118), and Zn (0.118–10.63) mg L^−1^. Table 3 shows the average concentrations of heavy metals in the water samples, highlighting the limits set by World Health Organization (WHO) [24] and Philippine National Standard for Drinking Water (PNSDW) [25]. The concentrations in SW and GW of Cd (0.009 and 0.030 mg L^−1^), Pb (0.011 and 0.042 mg L^−1^), and Mn (0.608 and 0.585 mg L^−1^) respectively, exceeded the maximum permissible limit (MPL) prescribed by both international and national regulatory bodies [24,25]. Likewise, Cr (0.051 mg L^−1^) and Zn (3.163 mg L^−1^) levels in GW samples both exceeded the MPL. Concentrations of Cr and Zn in SW, Cu, Fe, and Ni in both SW and GW were below MPL. Concentration of six (6) among the eight (8) metals (Cd, Cr, Fe, Pb, Mn, and Zn) exceeded the PNSDW limits in the GW of Torrijos, while five (5) did so in Sta. Cruz. The distribution trend of metal concentration in water is shown in Table 4. It illustrates that Mn had the highest concentration in SW while Zn in GW all across municipalities.

### 3.2. Heavy Metals Concentrations in Crustaceans and Tilapia

**Figure 2:**
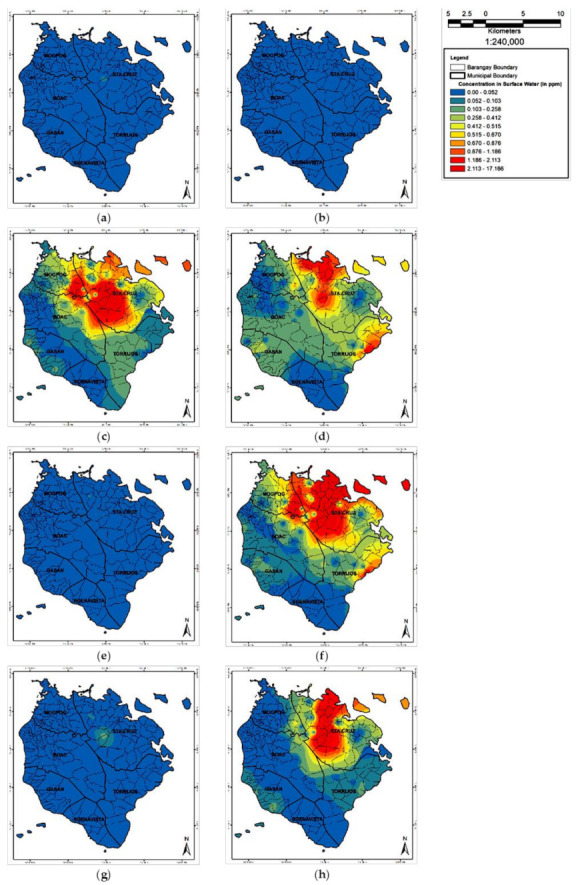
Surface water spatial distribution of metals concentrations in Marinduque: (**a**) Cd, (**b**) Cr, (**c**) Cu, (**d**) Fe, (**e**) Pb, (**f**) Mn, (**g**) Ni, and (**h**) Zn.

**Figure 3:**
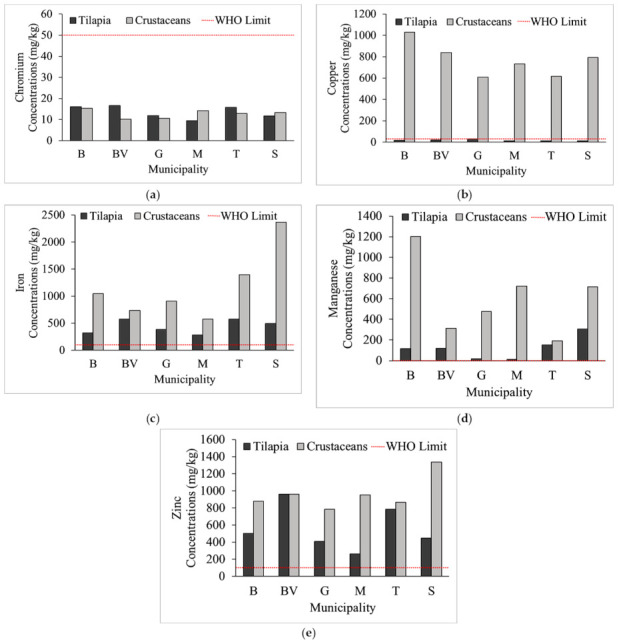
The concentrations of tilapia and crustaceans per municipality of the island province. Note: The red horizontal line represents the maximum permissible limit (MPL) set by WHO. The vertical bars are the concentration of metals in tilapia and crustaceans. The concentration of Cr (**a**) in both tilapia and crustaceans was below MPL. The concentration of Cu in crustaceans (**b**) was beyond the WHO MPL However, the Cu concentration in tilapia was below WHO MPL. The Fe concentration (**c**) in both tilapia and crustaceans was beyond MPL in all six municipalities. The Mn concentration (**d**) in both tilapia and crustaceans was beyond WHO MPL. Mn concentration in crustaceans collected from Gasan and Mogpog recorded the lowest concentration among the six municipalities. The municipality of Boac recorded the highest Mn concentration in crustaceans. The Zn concentration (**e**) in both tilapia and crustaceans was beyond the MPL in all six municipalities. Sta. Cruz municipality recorded the highest concentration in the island province for iron and zinc. Boac municipality recorded the highest concentration for copper and manganese.

Figure 2 illustrates the spatial concentration distribution of these eight metals in SW with tilapia and crustaceans. The concentrations of the heavy metals in crustaceans (*M. lar*, *M. placidulum*) and tilapia (*O. niloticus* and *C. striata*) in wet weight (w.w.) are enumerated in Table 5. Recorded values for the six municipalities are shown in Figure 3. The red horizontal line represents the maximum permissible limit set by the international regulatory bodies [18,26,27,28]. The metals concentrations in crustaceans and tilapia recorded the following: Cr = 44.61–79.42, Cu = 53.39–4898, Fe = 1344–1.129 × 10^4^, Mn = 53.33–5731, and Zn = 1266–6362 in dry weight (d.w.) basis, respectively. Concentrations of Cd, Ni, and Pb were below the instrument detection limits; hence, were reported as non-detected (ND) analytes. The average concentration of Zn was 562.2 and 963.8 mg kg^−1^ w.w. in tilapia and crustaceans, respectively. The Cu in crustaceans (770.2 mg kg^−1^ w.w.) exceeded the MPL set by international regulatory bodies (highlighted numbers).

### 3.3. Human Health Risk of Heavy Metals by Ingestion

**Figure 4:**
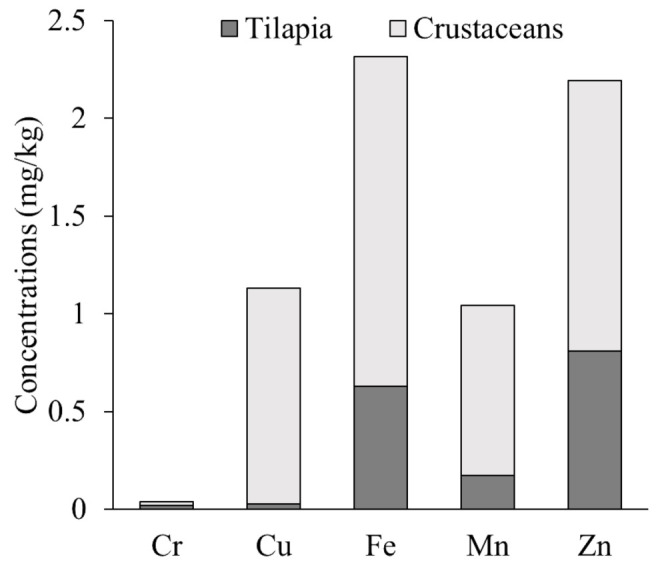
Contribution of crustaceans and tilapia to the mean EDI.

The EDI of metals through crustaceans and tilapia by the population in Marinduque is presented in Table 6. The crustaceans contributed more than tilapia to the population EDI as illustrated in Figure 4. The metals of concern in freshwater crustaceans and tilapia were Cu, Fe, Mn, and Zn.

The computed EDI range for the SW and GW samples were Cd (2.308 × 10^−5^–4.138 × 10^−3^), Cr (7.184 × 10^−5^–4.714×10^−3^), Cu (3.617 × 10^−5^–1.022 × 10^−1^), Fe (2.082 × 10^−4^–4.459 × 10^−1^), Pb (1.674 × 10^−5^–4.868 × 10^−3^), Mn (5.671 × 10^−4^–1.021 × 10^−1^), Ni (3.846 × 10^−5^–4.901 × 10^−3^), and Zn (1.157 × 10^−5^–4.427 × 10^−1^). The SW and GW contributions to the human mean daily intake of the heavy metals revealed that SW contributed Cu and Mn concentrations more than the GW; however, the later contributed more Fe and Zn.

The potential health hazard of the toxicants was interpreted based on the values of the THQ and HHI. Table 7 shows the calculated potential HHI of the heavy metals found in crustaceans, tilapia, SW, and GW. Ingestion of the SW and GW samples recorded to have THQ values ranged from 2.308 × 10^−2^–4.138 × 10^0^ Cd, 4.789 × 10^−5^–3.143 × 10^−3^ Cr, 6.144 × 10^−2^–2.556 × 10^0^ Cu, 2.975 × 10^−4^–6.370 × 10^−1^ Fe, 4.186 × 10^−3^–1.217 × 10^0^ Pb, 4.051 × 10^−3^–7.292 × 10^−1^ Mn, 1.641 × 10^−3^–2.451 × 10^−1^ Ni, and 3.858 × 10^−5^–1.476 × 10^0^ Zn. The HHI values for SW and GW in Mogpog (1.001 × 10^0^ and 2.607 × 10^0^, respectively) and in Sta. Cruz (5.341 × 10^0^ and 5.013 × 10^0^, respectively) were greater than 1. Further, GW samples from Torrijos had HHI value of 6.714 × 10^0^, which was way above 1. The HHI values greater than 1 pose a potential high health risk to human population.

Consumption of crustaceans and tilapia on a daily basis of 87.1 g leads to a potential accumulation of 0.009–0.016 Cr, 0.403–36.98 Cu, 0.580–4.870 Fe, 0.115–12.36 Mn, and 1.264–6.403 Zn. Fish and crustacean species have HHI > 1. Summing up the THQs of the targeted metals pointed to an adverse health effects of the exposed population. It was recorded that the HHI values for tilapia (2.381–7.784) were much lower than the HHI values for the crustaceans (31.18–55.73). This means the ingestion of crustaceans was more hazardous than tilapia.

The likelihood of the concerned receptors developing cancer due to Cr, Cd, Ni, and Pb exposure (metals are carcinogenic) was evaluated using EDI and CSF to carry out CRA. This evaluation was carried out based on the results of HHI that recorded greater than one. It showed that the average population life time cancer risk associated with crustaceans and tilapia consumption (0.007–0.012) and ingestion of SW and GW (1.423 × 10^−7^–2.607 × 10^−2^) would result to approximately 1 case per 100,000 population. As per the U.S. Environmental Protection Agency [16], the value of 10^−5^ is an acceptable lifetime carcinogenic risk. However, it should be noted that the PER of the island for metals in crustaceans and tilapia was high. Hence, the cumulative effects of trace metals may lead to chronic poisoning as a result of long-term exposure.

### 3.4. The Cumulative Potential Ecological Risks (PER)

**Figure 5:**
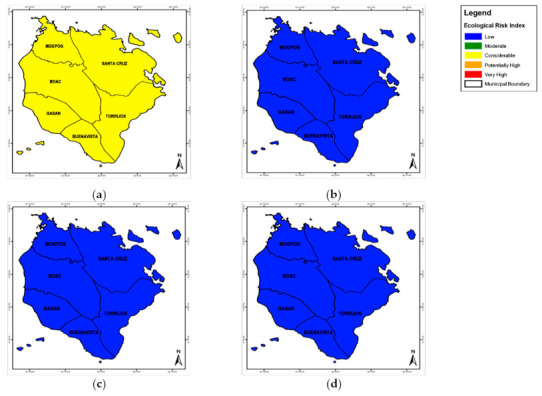
The spatial distribution of pERI at municipal level by the elevated metals concentration in (**a**) surface water, (**b**) groundwater, (**c**) crustaceans, and (**d**) tilapia.

The pERI at municipal level is illustrated by Figure 5 with areas in blue with the highest pERI. Tilapia, SW, and GW had pERI values lower than 150, which means low potential ecological risk. The pERI by crustaceans and tilapia in six municipalities recorded a range of 0.991–1.765 Cr and 7.233–36.36 Zn. This is less than forty (40), which means low potential ecological risk. Also, the pERI of Cu by tilapia recorded 5.339–14.28, which indicated low PER. However, pERI of Cu by crustaceans for Gasan and Torrijos recorded 290.1 and 293.0, respectively which means considerable risk. Whereas, the pERI Cu for Boac (498.8), Buenavista (399.5), Mogpog (349.3), and Sta. Cruz (377.9), which means high potential risk. The pERI of Cu by crustaceans collected from Gasan and Torrijos were greater than 160, which means considerable risk. While, the pERI of Cu collected from the other four municipalities exceeded three hundred (300), which means high potential ecological risk. The calculated pERI for the heavy metals found in crustaceans, tilapia, SW, and GW were tabulated in Table 8. The cumulative pERI, which is the summation of coefficients $E_{r}^{i}$, is low for tilapia (13.56–37.81), SW (1.587 × 10^−6^–1.230 × 10^0^), and GW (1.941 × 10^−2^–3.030 × 10^0^). Cumulative pERI for crustaceans (312.5–515.4) was considerable. Elevated concentration of Cr, Cu ad Zn was common in crustaceans and tilapia.

The potential ecological risk coefficients order of identified metals in crustaceans, tilapia, SW, and GW is summarized in Table 9. Only Zn had a potential ecological risk coefficient for Buenavista, though it recorded elevated concentrations for Fe and Mn. This was due to the absence of the standard pre-industrial reference level. The municipality of Sta. Cruz recorded PER order for SW as Cd > Cu > Zn > Pb > Cr. The collected GW samples had PER orders as follows: (a) Boac: Cd > Cu > Cr > Pb > Zn, (b) Buenavista: Cd > Zn > Cr > Pb > Cu, (c) Gasan: Cd > Cu > Zn > Cr > Pb, (d) Mogpog: Zn > Cd > Cu > Pb > Cr, (e) Torrijos: Cd > Zn > Cu > Pb > Cr and (f) Sta. Cruz: Cd > Cu > Zn > Pb > Cr.

### 3.5. Relationship between the Human Health and the Environment

**Figure 6:**
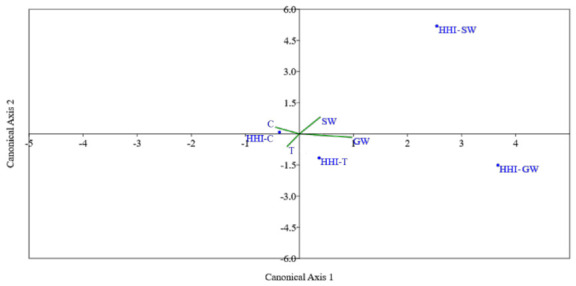
The canonical correspondence analysis (CCA) ordination of the ERI and HHI. Blue dots represent the HHI of crustaceans (HHI-CCA), tilapia (HHI-T), SW (HHI-SW), and GW (HHI-GW). The green lines represent the pERI of the crustaceans, tilapia, SW, and GW.

The calculated PERs were used as the environmental variables (synthetic gradients) for the CCA and results were displayed by an ordination diagram where the synthetic gradients are indicated by green lines and the calculated HHIs are illustrated by dots (Figure 6). The important variables were known by their correlations with the Canonical Axis 1 and Canonical Axis 2. The CCA revealed that the second canonical axis is negatively correlated with the PER of GW samples, while positively correlated with the PER of the crustaceans. These data denote that the HHI of crustaceans (HHI_C) were significantly affected by its PER. The CCA maximizes the predictive power of the synthetic gradients. The departed plots in Figure 6 of HHIs of the SW (HHI_SW) and GW (HHI_GW) illustrated that these two areas were not significantly affected. The total inertia of the CCA of 60.98% suggested that the HHIs could be predicted by the PERs and effect/s on the HHI were significant. Thus, the PERs associated with the crustaceans, tilapia, and water tell us the pattern of HHIs in the environment by the tilapia/curstacean and water samples.

The Pearson correlation analyses were utlized to examine the relationships between the levels of heavy metals in crustaceans, tilapia, and water samples. It had two significant variables (*r*, *p*) in the analysis. The *r* tells us a relationship between variables. Whereas, the *p* informs us if the relationship is statistically significant. The *p*-value (*p*) denotes the correlation is significant if values ranged at the 0.01 and 0.05 bracket. Lower p-value denotes that correlation is statistically significant, whereas higher p-value denotes the inverse. The Cr in tilapia was more strongly positively related to Zn, *r* (11) = 0.726, *p* < 0.01, than to Fe, *r* (11) = 0.711, *p* < 0.01. Likewise, Fe was strongly positively related to Mn, *r* (11) = 0.700, *p* < 0.01. For the crustaceans, Cu was strongly positively related to Zn, *r* (11) = 0.683, *p* < 0.05. On the other hand, Pearson correlation analysis for the relationships between Cd, Cr, Cu, Fe, Pb, Mn, Ni, and Zn in the water samples showed significant positive correlation with other metals as enumerated in Table 10 and Table 11 Positive significant correlations between these various metals suggested that these metals have similar origin, mutual dependence, and identical behavior during transport [8,29,30].

## 4. Discussion

Detected concentration of some transition and post-transition metals; e.g., Cd, Cr, Cu, Fe, Mn, Ni, Pb, and Zn, recorded elevated values. Elevated concentration was also observed in the work at Dhaka [31], India [32], Congo [33], and Malaysia [34]. The spatial distribution of various metals concentration in inland water as illustrated by Figure 2 and elaborated in Table 3 showed that the municipality of Sta. Cruz and Torrijos, respectively, had the highest metals concentration among the six municipalities. Concentration of Mn was the highest in all SW samples, and Zn in GW water samples across the island. The GW discharges to SW as part of the hydrological cycle. Hence, the SW and GW are interconnected with each other and possess a relationship during transport of contaminants. The affinity and fate of Mn and Zn during transport was associated with gram-positive bacteria [35] and gram-negative bacteria for some transition metals [36]. Though these studies were experimental and laboratory scale, it could be figured out the similar behavior of these microorganisms in the macro environment. Some mining activities used microorganisms such as *Thiobacillus Ferrooxidans* bacteria for metals extractions or recovery from ore [37,38,39]. The elevated metals concentrations in SW and GW were associated to continuous subsurface flow of toxicants in inland water. This could be attributed to the two abandoned open mine pits [8,9] located at the higher elevation (Maguilaguila and Mt. Tapian) of the island, its geological porous media written in Section 2.1, and the elevated concentration of these transition metals in soil [40]. These environmental conditions are similar to the areas described in Ghana [41] and Bolivia [42], which have mining activities [41,42] and areas in proximity to industrial zones [31,32,33,34]. Elevated Zn and Mn concentration in SW was also recorded in the mining-affected areas in Bolivia [42].

Recorded concentration of Zn in both crustaceans and tilapia, and Cu in crustaceans exceeded the maximum permissible limit. Also, concentration of Cr in both tilapia and crustaceans exceeded the USEPA [18] limit. Metals concentrations in crustaceans was higher than in tilapia. Similar results were recorded in Indonesia [43], India [44], Senegal [45], Brazil [46], and Nigeria [47]. However, the recorded Cr concentration of this study was higher than the recorded values of the countries written above. Likewise, the concentration of Cu in shrimps was way above the limit set by WHO [27] and USEPA [18]. These results of metals concentration in crustaceans and tilapia were attributed to various factors. These factors are temporal tropical climate, annual mean temperature of 27 °C with no defined wet and dry season through the year, body size [48] (which affect uptake difference between crustaceans and fishes as illustrated in Figure 4), habitat, feeding nature, bioavailability, feeding efficiency [44], and availability of metals species. The metals concentration was compared with neighboring countries along the meridian (latitude), where the Philippines is located. The Cu concentration reported by India [6], Indonesia [43], Senegal [45], Brazil [46], and Singapore [49] was way below the recorded concentration values in this study. In the work of Giri and Singh [50] on the Subarnarekha River in India, the concentration range of metals in fish and shrimp (crustaceans) were lower than the concentration range of this study. Mn was not included in the scope of Giri and Singh [50]. This Subarnarekha river is also rich in minerals and hosts minerals–based industry with unplanned and unregulated mining activities [51]; hence, this condition was similar to the island of Marinduque in which presence of mining activities contribute to the elevated concentration of metals in water, freshwater crustaceans, and fish.

Long-term exposure to crustaceans, tilapia, and inland water in Marinduque may result to one cancer patient per 100,000 population, which according to USEPA [16] is acceptable. Crustaceans, tilapia, and inland water were only the three edible items in Marinduque considered in the calculation of cancer risk probability in this study. This means additional ingestion of other edibles with elevated metals concentration adds onto the health risks posed by crustaceans, tilapia, and water. This condition was also elaborated by the work of Anankumar et al. [48] and Orosun et al. [47], who showed that exposure to toxic and trace metals at higher concentrations will cause chronic health adverse effects to humans, including HIV prevalence [52], anemia [53], and mental health problems/cognitive impairment [54,55]. Further, ingestion of metals and metalloids in crops contribute to morbidity and/or mortality [55]. The highest cumulative PER areas based on inland water, crustaceans, and tilapia are the municipalities of Sta. Cruz, Torrijos, and Boac. The order of PER for GW and SW at Sta. Cruz were the same. This was attributed to the two abandoned open mine pits in Marinduque as this island sits on volcanic, igneous, and volcanic rocks [9]. Similar condition of exposure to mine tailings by the population in South Africa was elaborated by the work of Ngole-Jeme and Fantke [52]. Also, the positive correlation and affinity of transition and post-transition metals were attributed to a reducing environment such as deep abandoned open mine pit in which weathering is taking place—producing sulphides from acid rock drainage. These sulphides could carry metals during transport through porous media, which Marinduque has [9], such as Cd, Cr, Ni, Pb, and Zn, as illustrated in Table 3 and Table 4 and Figure 3 and Figure 4. This condition was also discussed by the Geological Survey of Sweden [30].

## 5. Conclusions

This study conducted sampling activities, laboratory analysis using ICP-OES, followed Hakanson Sedimentological Approach, employing CCA, IBM SPSS, Pearson correlation, and utilizing the GIS tool to provide information on the spatial distribution of metals concentration and potential ecological risks to the island province of Marinduque, Philippines. Inland water, crustaceans, and tilapia were samples used in the study. Eight metals (Cd, Cr, Cu, Fe, Mn, Ni, Pb, and Zn) were targeted in this study. Results showed that Mn concentration was highest in SW, while Zn was highest in GW among the other eight metals that were analyzed. The GW of the municipalities of Sta. Cruz and Torrijos recorded a larger area of elevated concentration. Also, the riverside barangays in Mogpog municipality had elevated concentration of Cd, Cr, Fe, Pb, and Mn in GW. The Mn had the highest concentration in SW. The Zn concentration was highest in GW. This condition (i.e., highest concentration of Mn in SW and Zn in GW) was across all six municipalities of the island province. The municipality of Sta. Cruz recorded the highest concentration of metals among the six municipalities of Marinduque. Elevated concentration of Zn was detected in both crustaceans and tilapia. Elevated Cu was detected in crustaceans only. The crustaceans recorded higher concentrations of the eight metals compared to tilapia. The municipality of Torrijos and Sta. Cruz recorded the highest pERI for GW among the six municipalities. The *O. niloticus* and *C. striata* had low pERI while considerable for *Macrobrchium species.* However, there is high potential health risk if consumed. The likelihood of the receptor to develop or acquire cancer due to exposure to the abovementioned transition metals was one case per 100,000 population (10^−5^). This was considered acceptable lifetime carcinogenic risk. Further, the result of the statistical analysis showed that the metals concentration in SW and GW suggested to have similar source/s, mutual dependence, and identical behavior during transport. The data in this study are useful in the improvement of existing policies and programs, and creating strategies for the sustainable development of the island province.

## Figures and Tables

**Table 1:** Gradation (critical) range for contamination factor, degree of contamination, risk coefficient, and index [17].

Potential Ecological Risks (PER)	$C_{f}^{i}$	*C_d_*	$E_{r}^{i}$	Ecological Risk Index (ERI)
Low	$C_{f}^{i}$ < 1	$C_{d}$ < 8	$E_{r}^{i}$ < 40	ERI < 150
Moderate	1 ≤ $C_{f}^{i}$ < 3	8 ≤ $C_{d}\;$< 16	40 ≤ $E_{r}^{i}$ < 80	150 ≤ ERI < 300
Considerable	3≤ $C_{f}^{i}$ < 6	16 ≤ $C_{d}\;$< 32	80 ≤ $E_{r}^{i}$ < 160	300 ≤ ERI< 600
Potentially high	-	-	160 ≤ $E_{r}^{i}$ < 320	-
Very high	$C_{f}^{i}$ ≥ 6	$C_{d}$ ≥ 32	$E_{r}^{i}$ ≥ 320	ERI ≥ 600

**Table 2:** Background reference values $C_{n}^{i}$ and $T_{r}^{i}$ [17].

Heavy Metal	$C_{n}^{i},\;\mathrm{mg}\;\mathrm{kg}{-}^{1}$	$T_{r}^{i}$
Cadmium (Cd)	1	30
Chromium (Cr)	90	2
Copper (Cu)	50	5
Lead (Pb)	70	5
Zinc (Zn)	175	1

**Table 3:** Average concentration of metals in water samples (mg L^−1^) with maximum permissible limit (MPL).

Location	Water Sample	Metal							
		Cd	Cr	Cu	Fe	Pb	Mn	Ni	Zn
Boac	SW	0.009	0.005	0.076	0.094	0.009	0.029	0.012	0.016
	GW	0.001	0.034	0.034	1.233	0.010	1.483	0.001	0.118
Buenavista	SW	-	-	-	0.005	-	0.014	-	-
	GW	0.001	0.017	0.001	0.482	0.002	0.098	0.001	0.385
Gasan	SW	0.013	0.007	0.059	0.204	0.012	0.093	0.012	0.062
	GW	0.001	0.018	0.121	0.750	0.002	0.304	0.001	1.600
Mogpog	SW	-	-	0.717	0.127	-	0.765	0.002	0.082
	GW	0.001	0.042	0.075	10.70	0.027	0.363	0.001	10.63
Torrijos	SW	0.002	0.002	0.115	0.355	0.011	0.299	0.006	0.067
	GW	0.099	0.113	0.115	2.565	0.117	0.479	0.118	5.011
Sta. Cruz	SW	0.032	0.016	2.454	0.846	0.032	2.450	0.043	1.651
	GW	0.079	0.081	0.113	0.691	0.093	0.783	0.094	1.240
	SW	±0.018	+0.010	±2.530	±0.597	±0.017	±2.070	±0.026	±1.404
*SD*	GW	±0.045	±0.041	±0.058	±9.375	±0.051	±1.532	±0.053	±10.428
**WHO** [24]		**0.003**	**0.050**	**2.000**	**3.000**	**0.010**	**0.050**	**0.070**	**3.000**
**PNSDW** [25]		**0.003**	**0.050**	**1.000**	**1.000**	**0.010**	**0.400**	**0.070**	**5.000**

Note: GW—groundwater; SW—surface water; SD—Standard Deviation; WHO—World Health Organization; PNSDW—Philippine National Standards for Drinking Water. Bold numbers are limits set by WHO and PNSDW per metal.

**Table 4:** Trend of metal concentration accumulation in water across the island.

Location	Water Sample	
	SW	GW
Boac	Mn > Cu > Zn > Fe > Cd > Pb > Cr > Ni	Zn > Fe > Mn > Cu > Cr > Pb > Cd > Ni
Buenavista	Mn > Fe	Zn > Fe > Mn > Cd > Cr > Ni > Cu > Pb
Gasan	Mn > Cu > Zn > Fe > Ni > Cd > Pb > Cr	Zn > Cu > Fe > Mn > Cr > Ni > Cd > Pb
Mogpog	Mn > Cu > Zn > Fe > Ni	Zn > Fe > Mn > Cu > Pb > Cr > Ni > Cd
Torrijos	Mn > Fe > Cu > Zn > Pb > Ni > Cd > Cr	Zn > Fe > Mn > Cr > Cu > Ni > Pb > Cd
Sta. Cruz	Mn > Zn > Cu > Fe > Ni > Pb > Cd > Cr	Zn > Fe > Mn > Cr > Cu > Ni > Pb > Cd

**Table 5:** Concentrations of toxic metals in crustaceans and tilapia (mg kg^−1^ w.w.) with MPL.

Biota	Metal							
	Cd	Cr	Cu	Fe	Pb	Mn	Ni	Zn
Tilapia	ND	13.59	17.72	438.8	ND	121.1	ND	**562.2**
SD	-	±3.19	±10.17	+174.1	-	±157.1	-	±315.4
Crustaceans	ND	12.74	770.2	1172	ND	603.5	ND	963.8
SD	-	±3.64	±239.9	±788.9	-	±602.6	-	±271.7
**FAO** [26]	**0.500**	-	**30.00**	-	**0.500**	-	-	**40.00**
**WHO** [27]	**1.000**	**50.00**	**30.00**	100	**2.000**	1	**0.500–1.000**	**100.0**
**USEPA** [18]	**2.000**	**8.000**	**120.0**	-	**4.000**	-	-	**120.0**
**EC** [28]	**0.050**	-	-	-	**0.300**	-	-	**120.0**

Note: SD—Standard Deviation; FAO—Food and Agricultural Organization; WHO—World Health Organization; US EPA—United States Environmental Protection Agency; EC—European Commission. Bold numbers are concentration limits set by FAO, WHO, USEPA, and EC.

**Table 6:** The estimated daily intake (EDI) of metals (mg kg^−1^ day^−1^) through crustaceans and tilapia.

Location	Biota	Metal							
		Cd	Cr	Cu	Fe	Pb	Mn	Ni	Zn
Boac	Tilapia	-	0.023	0.026	0.462	-	0.165	-	0.724
	Crustaceans	-	0.022	1.479	1.508	-	1.730	-	1.266
Buenavista	Tilapia	-	0.024	0.030	0.826	-	0.173	-	1.382
	Crustaceans	-	0.015	1.206	1.057	-	0.448	-	1.381
Gasan	Tilapia	-	0.017	0.043	0.555	-	0.028	-	0.588
	Crustaceans	-	0.015	0.876	1.310	-	0.685	-	1.127
Mogpog	Tilapia	-	0.013	0.016	0.406	-	0.016	-	0.382
	Crustaceans	-	0.020	1.055	0.824	-	1.039	-	1.372
Torrijos	Tilapia	-	0.023	0.019	0.824	-	0.221	-	1.130
	Crustaceans	-	0.019	0.887	2.006	-	0.274	-	1.247
Sta. Cruz	Tilapia	-	0.017	0.019	0.713	-	0.441	-	0.644
	Crustaceans	-	0.019	1.141	3.409	-	1.030	-	1.921

**Table 7:** Potential health hazard index (HHI) by ingestion of aquatic resources from Marinduque.

Location	HHI			
	Crustaceans	Tilapia	SW	GW
Boac	55.73	4.920	6.027 × 10^−1^	6.921 × 10^−1^
Buenavista	39.48	7.784	4.387 × 10^−3^	1.576 × 10^−1^
Gasan	32.43	4.040	7.880 × 10^−1^	5.262 × 10^−1^
Mogpog	39.55	2.381	1.001 × 10^0^	2.607 × 10^0^
Torrijos	31.18	7.005	4.504 × 10^−1^	6.714 × 10^0^
Sta. Cruz	47.17	6.806	5.341 × 10^0^	5.013 × 10^0^

**Table 8:** The potential ecological risk index (pERI) in aquatic resources from Marinduque.

Location	pERI			
	Crustaceans	Tilapia	SW	GW
Boac	515.4	24.03	2.889 × 10^−1^	2.217 × 10^−2^
Buenavista	426.7	37.81	1.587 × 10^−6^	1.941 × 10^−2^
Gasan	312.5	26.66	3.880 × 10^−1^	3.840 × 10^−2^
Mogpog	376.7	13.56	7.215 × 10^−2^	8.774 × 10^−2^
Torrijos	318.9	29.24	7.632 × 10^−2^	3.030 × 10^0^
Sta. Cruz	415.7	19.74	1.230 × 10^0^	2.387 × 10^0^

**Table 9:** Trend of potential ecological risk coefficient in crustaceans, tilapia, SW, and GW.

Location	Samples			
	Crustaceans	Tilapia	SW	GW
Boac	Cu > Zn > Cr	Zn > Cu > Cr	Cd > Cu > Pb > Cr > Zn	Cd > Cu > Cr > Pb > Zn
Buenavista	Cu > Zn > Cr	Zn > Cu > Cr	Zn	Cd > Zn > Cr > Pb > Cu
Gasan	Cu > Zn > Cr	Cu > Zn > Cr	Cd > Cu > Pb > Zn > Cr	Cd > Cu > Zn > Cr > Pb
Mogpog	Cu > Zn > Cr	Zn > Cu > Cr	Cu > Zn > Pb	Zn > Cd > Cu > Pb > Cr
Torrijos	Cu > Zn > Cr	Zn > Cu > Cr	Cd > Cu > Pb > Zn > Cr	Cd > Zn > Cu > Pb > Cr
Sta. Cruz	Cu > Zn > Cr	Zn > Cu > Cr	Cd > Cu > Zn > Pb > Cr	Cd > Cu > Zn > Pb > Cr

**Table 10:** Correlation analysis of heavy metals in surface water samples using International Business Machine(IBM) Statistical Package for Social Sciences (SPSS), *N* = 62.

Metal	Cd	Cr	Cu	Fe	Pb	Mn	Ni	Zn
Cd		0.968 **	0.452 **	0.243	0.940 **	0.251 *	0.786 **	0.439 **
Cr			0.368 **	0.241	0.896 **	0.211	0.705 **	0.358 **
Cu				0.489 **	0.386 **	0.712 **	0.725 **	0.911 **
Fe					0.252 *	0.680 **	0.410 **	0.613 **
Pb						0.212	0.721 **	0.378 **
Mn							0.430 **	0.859 **
Ni								0.691 **
Zn								

** Correlation is significant at the 0.01 level (2-tailed). * Correlation is significant at the 0.05 level (2-tailed).

**Table 11:** Correlation analysis on heavy metals in groundwater samples using SPSS (*N* = 62).

Metal	Cd	Cr	Cu	Fe	Pb	Mn	Ni	Zn
Cd		0.840 **	0.169	−0.021	0.959 **	0.074	0.959 **	0.056
Cr			−0.027	0.174	0.881 **	0.008	0.786 **	0.142
Cu				0.005	0.091	0.591 **	0.371 **	0.127
Fe					0.204	0.056	−0.014	0.858 **
Pb						0.042	0.908 **	0.244
Mn							0.184	0.072
Ni								0.078
Zn								

** Correlation is significant at the 0.01 level (2-tailed).

## Data Availability

All data are contained in the manuscript.
